# Supplementary material for: The value of admission avoidance: cost-consequence analysis of one-year activity in a consolidated service
Source: Cost Eff Resour Alloc. 2024 Apr 15;22:30. doi: 10.1186/s12962-024-00536-1 (PMC11017527; doi:10.1186/s12962-024-00536-1)
Supplement: Supplementary file 1 — Supplementary Material 1 [file 12962_2024_536_MOESM1_ESM.docx]

# The Value of Admission Avoidance: Cost-Consequence Analysis of One-Year Activity in a Consolidated Service

*Hernandez C. et al (on-line supplementary material)*

**EXPANDED RESULTS – CHARACTERISTICS OF THE STUDY GROUPS**

**Table 1S** displays baseline data of the entire HaH-HA population (n=586), the HaH-HA group (n= 441) selected after Propensity Score Matching (PSM) and the corresponding Controls, conventional hospitalizations.

**Table 2S** informs on the distribution of main diagnostics, ICD-10 coding, in HaH-HA and Controls.

**Table 3S** provides data during the acute episode and during the 30-day period after discharge for the same datasets described in **Table 1S**.

**Table 4S** depicts information on operational costs of the service during the acute episode, as well as the community-based expenditure during the 30-day period after discharge following the same organization of the information indicated for **Tables 1S** and **3S**.

**Table 5S** summary description of the implementation process of the HaH service at HCP in two sequential periods: 2006-2015 and 2016-2018, following the Consolidated Framework for Implementation Research (CFIR).

**Figure 1S** depicts the mean scoring for the different items of the questionnaire on patients/careers satisfaction administered to the HaH-HA patients at discharge.

**TABLE 1S.** Characteristics of the study population (n=586) and study groups (n=441 each) after propensity score matching, before admission. Only the p-values ≤ 0.05 have been displayed.

|  | **Study Population (n=586)** | **HaH-HA (n=441)** | **p value** | **Controls (n=441)** | **p value** |
| --- | --- | --- | --- | --- | --- |
| **SOCIO-DEMOGRAPHICS** |  |  |  |  |  |
| Age (years), mean (SD)* | 72.12 (16.32) | 72.71 (16.3) |  | 73.94 (16.01) |  |
| Gender (male), n (%)* | 334 (57.00) | 250 (56.69) |  | 262 (59.41) |  |
| **USE OF HEALTH CARE RESOURCES** | | | | | |
| **Hospital resources in previous 12 months** |  | | |  | |
| Rate of all-cause emergency room visit, mean (SD) | 1.71 (1.14) | 1.63 (1.04) |  | 1.75 (1.26) |  |
| Rate of all-cause Hospital admissions, mean (SD)* | 1.69 (1.13) | 1.66 (1.22) |  | 1.62 (1.3) |  |
| Rate of planned admissions, mean (SD) | 1.40 (0.70) | 1.37 (0.72) |  | 1.40 (0.87) |  |
| Last visit (days) to outpatient clinic before admission, mean (SD) | 79.34 (88.24) | 85.98 (91.96) |  | 91.39 (94.39) |  |
| Last hospitalisation (days) before admission, mean (SD) | 190.26 (108.14) | 192.16 (108.75) |  | 175.22 (126.70) |  |
| Length of stay in days (total days per year), mean (total) | 11.51 (2129) | 11.48 (1538) |  | 11.49 (1333) |  |
| Intensive care unit stays, n (%) | 27 (8.7) | 19 (8.5) |  | 18 (9.6) |  |
| Outpatient visits, mean (SD) | 6.41 (7.46) | 5.99 (7.19) |  | 5.45 (5.69) |  |
| **Hospital resources in previous 7 days** |  | | |  | |
| Outpatient visits, mean (SD) | 1.2 (0.55) | 1.11 (0.42) |  | 1.14 (0.42) |  |
| **Healthcare costs across tiers in previous year** |  | | |  | |
| € per year, mean (SD)* | 6,664.41 (8,738.58) | 5,627.06 (8118.86) |  | 6,543.05 (6868.5) |  |
| **MULTIMORBIDITY & SEVERITY** | | | | | |
| GMA scoring , mean (SD)* | 26.55 (15.82) | 24.95 (15.17) |  | 25.09 (14.51) |  |
| GMA category, n (%) |  | | |  | |
| *Tier 1 < P_50_* | 16 (3.63) | 6 (1.36) |  | 8 (1.81) |  |
| *Tier 2 [P_50_ - P_80_)* | 47 (10.66) | 31 (7.03) |  | 30 (6.8) |  |
| *Tier 3 [P_80_-P_95_)* | 83 (18.82) | 97 (22.00) |  | 69 (15.65) |  |
| *Tier 4 [P_95_-P_99_)* | 119 (26.98) | 83 (18.82) |  | 117 (26.53) |  |
| *Tier 5 ≥ P_99_* | 321 (72.79) | 224 (50.79) |  | 217 (49.21) |  |

**Legend.** CCA, Cost Consequence Analysis; Study population: all patients included in Hospital at Home-Hospital Avoidance (HaH-HA); HaH-HA, corresponds to the study population after propensity score matching (PSM); Controls, conventional hospitalizations, after PSM; GMA, Adjusted Morbidity Groups scoring; * Matching variables.

**Table 2S.** Distribution of main diagnosis at discharge.

| **Disease Category** | | **Study Group** | |
| --- | --- | --- | --- |
| **ICD 10** | **ICD Description** | **HaH** | **Controls** |
| ***Urinary tract infection*** | | **117** | **117** |
| **N10** | *Acute tubulo-interstitial nephritis* | 26 | 15 |
| **N17** | *Acute kidney failure* | 1 | 12 |
| **N39** | *Other disorders of urethra and urinary tract* | 70 | 70 |
| **N41** | *Inflammatory diseases of prostate* | 17 | 8 |
| **N45** | *Orchitis and epididymitis* | 3 | 12 |
| ***Chronic lower respiratory diseases*** | | **67** | **67** |
| **J42** | *Chronic bronchitis* | 12 | 15 |
| **J43** | *Emphysema* | 7 | 9 |
| **J45** | *Asthma* | 7 | 7 |
| **J47** | *Bronchiectasis* | 9 | 14 |
| **J44** | *Other chronic obstructive pulmonary disease* | 32 | 22 |
| ***Pneumonia*** | | **55** | **55** |
| **J12** | *Viral pneumonia* | 0 | 2 |
| **J13** | *Pneumococcal pneumonia* | 4 | 9 |
| **J15** | *Bacterial pneumonia, not elsewhere classified* | 1 | 7 |
| **J18** | *Pneumonia, unspecified organism* | 50 | 37 |
| ***Acute lower respiratory infection and other respiratory disorders*** | | **46** | **46** |
| **J96** | *Other diseases of lung* | 3 | 6 |
| **J98** | *Other diseases of respiratory system* | 8 | 16 |
| **J20** | *Acute bronchitis* | 2 | 3 |
| **J22** | *Unspecified acute lower respiratory infection* | 32 | 18 |
| **J84** | *Other interstitial pulmonary diseases* | 1 | 3 |
| ***Hearth failure (I50)*** | | **37** | **37** |
| ***Infections of the skin and subcutaneous tissue*** | | **33** | **33** |
| **L02** | *Cutaneous abscess, furuncle and carbuncle* | 1 | 1 |
| **L03** | *Cellulitis and acute lymphangitis* | 29 | 29 |
| **S81** | *Open wound of knee and lower leg* | 1 | 0 |
| **S70** | *Superficial injury of hip and thigh* | 0 | 1 |
| **L97** | *Chronic ulcer of skin* | 2 | 2 |
| ***Flu (J10,J09,J11)*** | | **25** | **25** |
| ***Symptoms, injury and poisoning*** | | **16** | **16** |
| **R68** | *General symptoms* | 3 | 1 |
| **R78** | *Find of drugs and other substnces, not normally found in blood* | 10 | 5 |
| **T82** | *Complications of cardiac and vascular prosth dev/grft* | 0 | 3 |
| **T81** | *Complications of procedures, not elsewhere classified* | 0 | 4 |
| **R50** | *Fever of unknown origin* | 1 | 3 |
| **T83** | *Complications of genitourinary prosth dev/grft* | 1 | 0 |
| **T85** | *Complications of internal prosth dev/grft* | 1 | 0 |
| ***Hipetensive diseases and other heart diseases*** | | **15** | **15** |
| **I11** | *Hypertensive heart disease* | 1 | 0 |
| **I13** | *Hypertensive heart and chronic kidney disease* | 4 | 0 |
| **I21** | *Acute myocardial infarction* | 1 | 5 |
| **I20** | *Angina pectoris* | 0 | 1 |
| **I26** | *Acute pulmonary heart disease* | 3 | 4 |
| **I48** | *Cardiac dysrhythmias* | 1 | 4 |
| **I82** | *Other venous embolism and thrombosis* | 5 | 1 |
| ***Pneumonitis caused by bronchial aspiration (J69)*** | | **13** | **13** |
| ***Neutropenia and anemia (D61, D70)*** | | **5** | **5** |
| ***Infections (A41, A09, B97)*** | | **2** | **2** |
| ***Malignant neoplasms (C34, C67)*** | | **2** | **2** |
| ***Other*** | | **8** | **8** |
| **K05** | *Gingivitis and periodontal diseases* | 3 | 2 |
| **H81** | *Disorders of vestibular function* | 1 | 0 |
| **J03** | *Acute tonsillitis* | 1 | 0 |
| **K62** | *Other diseases of anus and rectum* | 1 | 1 |
| **K85** | *Acute pancreatitis* | 1 | 5 |
| **M86** | *Osteomyelitis* | 1 | 0 |

**Legend.** (ICD-10-CM) International Classification of Diseases.

**TABLE 3S.** Characteristics of the acute episode and main outcomes for the entire population of HaH-HA and for the two study groups (n=441 each). Only the p-values ≤ 0.05 have been displayed.

|  | **Study Population (n=586)** | **HaH-HA (n=441)** | **p value** | **Controls (n=441)** | **p value** |
| --- | --- | --- | --- | --- | --- |
| **Total length of stay (days), mean (SD)** | 8.09 (4.88) | 7.89 (4.37) |  | 7.37 (6.17) |  |
| **Case Mix Index** | 0.68 | 0.69 |  | 0.73 |  |
| **Use of resources during Hospital Avoidance** |  | | |  | |
| All-cause Emergency Room visits, n (%) | 11 (1.87) | 6 (1.36) |  | N/A |  |
| All-cause In-Hospital re-admissions, n (%) | 28 (4.75) | 18 (4.08) |  | N/A |  |
| **Mortality during episode, n (%)** | 0 (0) | 0 (0) |  | 19 (4.31) | **N/A** |
| **Outcomes at 30 days after discharge** |  | | |  | |
| All-cause Emergency Room visits, n (%) | 36 (6.14) | 28 (6.35) |  | 34 (8.06) | **.032** |
| All-cause Hospital admissions |  | | |  | |
| Unplanned Hospital admissions, n (%) | 37 (6.31) | 24 (5.44) |  | 23 (5.45) |  |
| Planned admissions, n (%) | 19 (3.24) | 13 (2.95) |  | 10 (2.37) |  |
| Mortality, n(%) | 7 (1.19) | 7 (1.59) |  | 7 (1.66) |  |

**Legend.** CCA, Cost Consequence Analysis; Study population: all patients included in Hospital at Home-Hospital Avoidance (HaH-HA); HaH-HA, corresponds to the study population after propensity score matching (PSM); Controls, conventional hospitalizations, after PSM.

**Table 4S.** Operational Cost (in €) during the acute episode and expenses during 30-days after discharge for the entire population and the two study groups. Only the p-values ≤ 0.05 of the category totals have been displayed.

|  | **Study Population (n = 586)** | **HaH-HA (n = 441)** | **Controls (n = 441)** | **p value** |
| --- | --- | --- | --- | --- |
| **STAFF** | | | | |
| Department Head | 21,637 | 16,770 | 20,032 | **<**.001 |
| Nursing co-ordinator | 19,457 | 15,080 | 23,194 |  |
| Physicians | 127,022 | 98,415 | 104,983 |  |
| Resident Physicians | 0 | 0 | 101,166 |  |
| Registered nurses | 293,620 | 227,489 | 238,948 |  |
| Nursing assistants | 0 | 0 | 152,070 |  |
| Physiotherapists | 0 | 0 | 11,690 |  |
| Social workers | 0 | 0 | 11,690 |  |
| Secretary | 31,561 | 24,461 | 14,853 |  |
| **Staff** | **493,298** | **382,215** | **678,625** |  |
| **PHARMACOLOGICAL TREATMENT and NON-PHARMACOLOGICAL** | | | | |
| Anti-infective therapy | 59,694 | 36,296 | 27,488 |  |
| Other pharmacological treatment | 8,187 | 5,927 | 25,087 |  |
| Oxygen Therapy *(new prescription)* | 4,470 | 3,909 | N/A |  |
| Nebulizer Therapy *(new prescription)* | 2,515 | 2,314 | N/A |  |
| **Treatment** | **74,865** | **48,445** | **52,575** |  |
| **CONSUMABLES** | | | | |
| **Consumables** | **18,455** | **13,887** | **39,200** | <.001 |
| **DIAGNOSTIC TESTS** | | | | |
| Laboratory tests | 8,067 | 5,895 | 29,378 | <.001 |
| Diagnostic images | 4,706 | 3,190 | 25,194 |  |
| **Diagnostic Tests** | **12,773** | **9,085** | **54,572** |  |
| **STAFF TRANSPORTATION** | | | | |
| **Staff Transportation** | **21,286** | **16,017** | **N/A** | N/A |
| **CATERING** | | | | |
| **Catering** | **N/A** | **N/A** | **65,519** | N/A |
| **STRUCTURE** | | | | |
| **Structure** | **7,448** | **5,636** | **66,787** | <.001 |
| **TOTAL** | **628,126** | **475,286** | **957,279** | <.001 |
| **Transitional Care (30d after discharge)** | **515,094** | **337,360** | **451,078** | <.001 |

**Legend.** Study population: all patients included in Hospital at Home-Hospital Avoidance (HaH-HA); HaH-Ha, corresponds to the study population after propensity score matching (PSM); Controls, conventional hospitalizations, after PSM.

**Table 5S. Description of the implementation strategy following the CFIR approach** [1]**.**

| **CONSTRUCTS** | **Implementation at HCB (2006-2015)** | **Expansion at health-district level, AIS-BE (2016-2018)** | **Key recommendations** |
| --- | --- | --- | --- |
| **Intervention characteristics** |  |  |  |
| Intervention Source | Internally developed | Internally developed | -Hospital at home as an integrated care service  -Core components:  1) Hospital avoidance or early discharge; 2) Hospital-based professionals  3) Service workflow defined  4) Define target patients’ profiles  5) Transitional care strategies  -Adaptability of non-core components is required.  -Continuous quantitative & qualitative build-in evaluation is needed |
| Evidence Strength & Quality | Results from internal research [2, 3] | Results from period 1[4] |  |
| Relative Advantage | -Patients & caregivers: satisfaction  -Hospital-based professionals: satisfaction, organizational & clinical results  -Managers: health outcomes & cost containment | -Identical to period 1 +  -Health-district care providers endorse scale-up of the program  -Community-based professionals: mixed feelings *(acknowledge advantage, but competitive issues emerge)* |  |
| Adaptability | Core components: i) Hospital-based teams; ii) Hospital avoidance or early discharge; iii) Workflow defined & patient profiles; iv) Structure to support the workflow.  Adaptable components: i) Any other aspect | Core components:  -Identical to period 1 +  -Appropriate training & QA program  Adaptable components  -Any other aspect |  |
| Trialability | -Results from internal research [5-9]  -A building-blocks strategy with stepwise progression of deployment with continuous evaluation [8] | -Idem, as reported in the current manuscript |  |
| Complexity | High complexity process requiring coordination of: i) Clinical protocols; ii) Redefine tasks & roles; iii) Home-based logistics; iv) Digital support; v) Professionals’ training; vi) Information for patients; vi) Coordination among providers; vii) Reimbursement modalities | - Identical to period 1 |  |
| Design Quality & Packaging | -Key elements: i) Explanation of the intervention to patients; and ii) Home-based logistics | - Identical to period 1 |  |
| Cost | -Implementation costs were covered by efficiencies generated without allocation of a specific budget for this purpose  -Operational costs were calculated following an analytical approach [3, 4] showing health-value generation | -Expansion costs were covered by a new financial structure agreed with the single public payer  -Operational costs reported in the current paper. Cost-effectiveness confirmed |  |
| **OUTER SETTING** | | | |
| Patients’ needs & resources | -Alignment with PRISM (Patient-centered care): i) patient choices & barriers taken into account and solved; ii) transitional care after discharge implemented; iii) Lean strategy minimizing costs; and, iv) patients’ accessibility, satisfaction & opinions considered and taken as inputs to improve. | - Identical to period 1 +  -Patient experience program at HCB [10] | -Patient-centered orientation should be a core trait  -Networking across experiences enriches the programs  -Site customization is required to minimize potential negative impacts of external factors |
| Cosmopolitanism | -Build-up as a functional integration of healthcare providers at district level (AIS-BE) [12]  -Progress through continuous interactions among regional providers [11]  -Leading role at EU level (four stars reference site EIP-AHA) [13, 14] | -Further progress both at local *(regional consensus on home hospitalization driven by the single-public payer)* and EU levels [15] |  |
| Pier pressure | - Support from the single-public payer and internal managers  - Moderate transient resistances from professionals from primary care and other internal clinical units  -Expansion beyond HCB limited by reimbursement modalities to other health-district provider organizations | -Changes in reimbursement modalities facilitated expansion to other providers in the health district.  -Previous resistances disappeared |  |
| External Policy & Incentives | - Implementation was an internal decision with weak external support.  - Program should be considered as a learning experience  - EU funding provided additional financial support [16-18] | -Consolidation of the program at HCB fostered a specific mandate of the single-public payer to expand the program |  |
| **INNER SETTING** |  |  |  |
| Structure Characteristics | -Institutional traits favouring the driving role: i) dual mission (needs for tertiary care beds); ii) leading role of professionals in the management (efficiency is a must); iii) institutional choice towards continuum of care [19]; and, iv) digital transformation in place  -The vertical organization in clinical institutes was a relative barrier | -Consolidation of Institutional traits  -External support from the single-public payer | -Bottom-up/Top-down interactions are needed for success  -Key resources to generate and reinforce a positive climate change are needed |
| Networks & Communications | -High professional engagement + rather mature digital tools | - Identical to period 1 |  |
| Culture | -Mix of type 1 (team culture) and type 3 (entrepreneurial culture) with high engagement of professionals | -Transfer of HCB’s culture to the other AIS-BE’s providers |  |
| Implementation Climate | -Positive climate for entrepreneurship despite acknowledgment of some internal peer resistances  -Tension for change was limited to champions supported by management  -Initiative aligned with hospital values and rules. Change management required.  -The program was acknowledged as a high priority operation  -No incentives & rewards were planned, but the role of champions was acknowledged  - Goals & Feedback well defined accepted, evolving over time  -High involvement of professionals in the development of new interventions to improve patient care | -Maturity of implementation at HCB + -External factors *(financial incentives)* fostered a positive climate for a health district extension of the service  -Engagement of professionals from other providers was fostered by the prestige of the original team |  |
| Readiness for implementation | -Readiness for implementation at regional level [8, 19]and at HCB level  -High engagement of the leaders. Champion-driven initiative  -Creation of a specific unit at HCB  -Maturity of health information exchange platform | -Financial incentives facilitated expansion at health district level |  |
| **CHARACTERISTICS OF INDIVIDUALS** |  |  |  |
| Knowledge &Believes about the intervention | -Sustained positive perception of the intervention by both patients and professionals  - Acknowledgement of enhanced health outcomes and positive impact on costs  - Initial passive resistance of small sectors of professionals attenuated over time | -High level of acceptance at all levels  -Refinement of transitional care & vertical integration is under debate. | -Continuous monitoring of satisfaction levels and consideration of feedback from patients and professionals is highly recommended [20] |
| Self-efficacy | - The service generates novel interactions between patients and professionals that foster self-efficacy | -Identical to period 1 |  |
| Individual stage of change | -Progressive achievement of active engagement of stakeholders throughout the deployment process | - Identical to period 1 +  -Interestingly, consolidation of new professionals’ roles is triggering debates on organizational aspects |  |
| Individual identification with organization | -A culture of individual identification with unique traits of the HCB’s organization (health professionals’ involvement in management) facilitated implementation | - Identical to period 1 |  |
| Other personal attributes | -Tolerance, motivation, innovativeness, and learning style have been reinforced during the implementation process | - Identical to period 1 |  |
| **PROCESS** |  |  |  |
| Planning | -The deployment plan organized by blocks (specific patients’ groups associated to patients’ profiles of the clinical institutes at HCB) was designed and progressively implemented with adaptations considering feedback received from professionals and patients using different communication channels | - A multidisciplinary team used a PSDA (Plan-Do-Study-Act) methodology [17] during the period | -A building-blocks implementation strategy, with appropriate site customization prioritizing engagement, is required  -Continuous evaluation of results [21, 22] |
| Engaging | -Implementation leaders (champions) triggered and conducted deployment with support, and direct interactions, with HCB’s management  -A specifically trained group of professionals with high degree of commitment and a transversal multidisciplinary approach contributed to consolidation | - Identical to period 1 +  - As mentioned above, a debate on organizational aspects is currently open |  |
| Executing | -Previous experience with two reported RCTs [2, 7] helped to set the basis of the deployment plan, which was executed accordingly.  -Continuous monitoring and adaptability of the implementation process were key elements for successful adoption [4] | -Lessons learnt during the previous phase have defined the roots for the expansion phase beyond HCB |  |
| Reflecting & Evaluating | -Continuous quantitative & qualitative assessments were done and reported [4]  -Internal monthly meetings and periodic reporting to HCB’s executive committee were scheduled and done | - Identical to period 1 |  |

**Legend:**HCB: Hospital Clínic de Barcelona; AIS-BE:Area Integral de Salut Barcelona Esquerra

**Figure 1S – Results of the satisfaction questionnaire administered to HaH-HA patients/careers at discharge.**

**
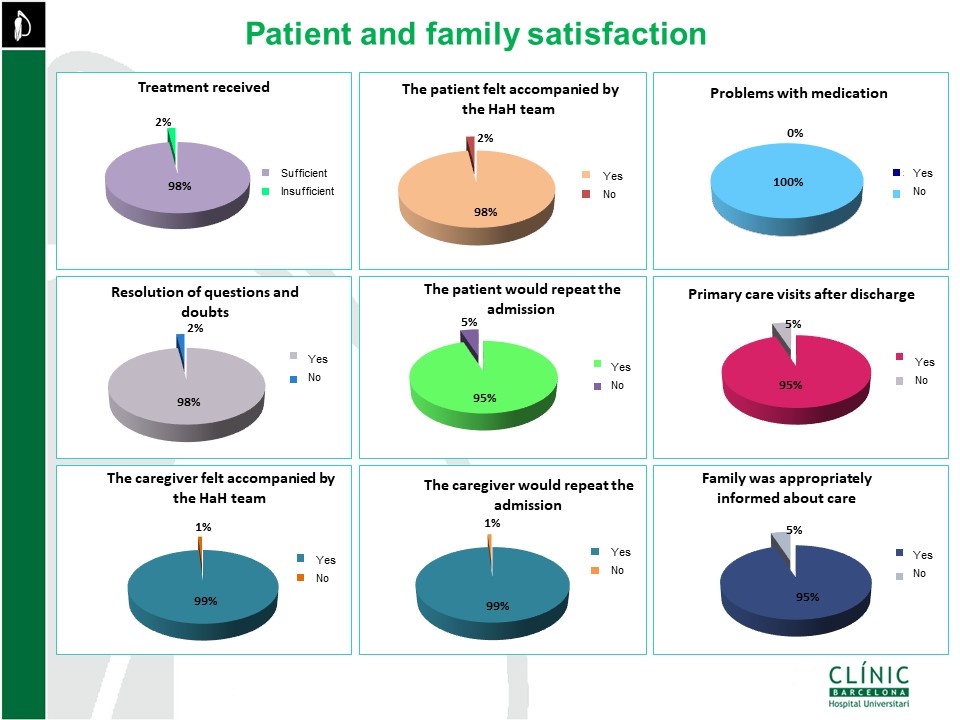
**

**REFERENCES**

1. Consolidated Framework for Implementation Research (CFIR). <https://cfirguide.org/constructs/>
2. [Hernández C](https://www.ncbi.nlm.nih.gov/pubmed/?term=Hernandez%20C%5BAuthor%5D&cauthor=true&cauthor_uid=12570110), et al. Home hospitalisation of exacerbated chronic obstructive pulmonary disease patients. [EurRespir J.](https://www.ncbi.nlm.nih.gov/pubmed/12570110) 2003 Jan;21(1):58-67.
3. [Puig-Junoy J](https://www.ncbi.nlm.nih.gov/pubmed/?term=Puig-Junoy%20J%5BAuthor%5D&cauthor=true&cauthor_uid=17221178)^,^ et al. The impact of home hospitalization on healthcare costs of exacerbations in COPD patients. [Eur J Health Econ.](https://www.ncbi.nlm.nih.gov/pubmed/17221178) 2007 Dec;8(4):325-32. Epub 2007 Jan 13.
4. [Carme Hernández](https://www.ncbi.nlm.nih.gov/pubmed/?term=Hern%26%23x000e1%3Bndez%20C%5BAuthor%5D&cauthor=true&cauthor_uid=30127696), et al. Implementation of Home Hospitalization and Early Discharge as an Integrated Care Service: A Ten Years Pragmatic Assessment. [Int J Integr Care](https://www.ncbi.nlm.nih.gov/pmc/articles/PMC6095082/). 2018 Apr-Jun; 18(2): 12.
5. CHRONIC - An Information Capture and Processing Environment for Chronic Patients in the Information Society. Contract nº IST-1999-12158
6. [C. Hernández](javascript:;), et al. The burden of chronic disorders on hospital admissions prompts the need for new modalities of care: A cross-sectional analysis in a tertiary hospital. *QJM: An International Journal of Medicine*, Volume 102, Issue 3, March 2009, Pages 193–202, <https://doi.org/10.1093/qjmed/hcn172>
7. [Casas A](https://www.ncbi.nlm.nih.gov/pubmed/?term=Casas%20A%5BAuthor%5D&cauthor=true&cauthor_uid=16611656), et al.Integrated care prevents hospitalisations for exacerbations in COPD patients. [EurRespir J.](https://www.ncbi.nlm.nih.gov/pubmed/16611656) 2006 Jul;28(1):123-30. Epub 2006 Apr 12.
8. [Hernández C](https://www.ncbi.nlm.nih.gov/pubmed/?term=Hern%C3%A1ndez%20C%5BAuthor%5D&cauthor=true&cauthor_uid=26034465), et al. Integrated care services: lessons learned from the deployment of the NEXES project. [Int J Integr Care.](https://www.ncbi.nlm.nih.gov/pubmed/26034465) 2015 Mar 30;15:e006. eCollection 2015 Jan-Mar.
9. [Hernández C](https://www.ncbi.nlm.nih.gov/pubmed/?term=Hern%C3%A1ndez%20C%5BAuthor%5D&cauthor=true&cauthor_uid=25856791), et al. Effectiveness of community-based integrated care in frail COPD patients: a randomised controlled trial. [NPJ Prim Care Respir Med.](https://www.ncbi.nlm.nih.gov/pubmed/25856791) 2015 Apr 9;25:15022. doi: 10.1038/npjpcrm.2015.22.
10. Caneiras C, et al. Patient Experience in Home Respiratory Therapies: Where We Are and Where to Go. J Clin Med 2019, 8(4), 555. Published Online First: 2019. doi:10.3390/jcm8040555
11. [Juan Carlos Contel](https://www.ncbi.nlm.nih.gov/pubmed/?term=Contel%20JC%5BAuthor%5D&cauthor=true&cauthor_uid=26150763), et al. Chronic and integrated care in Catalonia. [Int J Integr Care](https://www.ncbi.nlm.nih.gov/pmc/articles/PMC4491324/). 2015 Apr-Jun; 15: e025.
12. [Font D](https://www.ncbi.nlm.nih.gov/pubmed/?term=Font%20D%5BAuthor%5D&cauthor=true&cauthor_uid=27616964), et al. Integrated Health Care Barcelona Esquerra (Ais-Be): A Global View of Organisational Development, Re-Engineering of Processes and Improvement of the Information Systems. The Role of the Tertiary University Hospital in the Transformation. [Int J Integr Care.](https://www.ncbi.nlm.nih.gov/pubmed/?term=Font%2C+D%2C+et+al.+Int+J+Integr+Care.+2016+May+23%3B16(2)%3A8) 2016 May 23;16(2):8. doi: 10.5334/ijic.2476.
13. EIP-AHA. European Innovation Partnership on Active and Healthy Ageing. Available from: <https://ec.europa.eu/eip/ageing/news/november-edition-eip-aha-newsletter_en>
14. Best practice implementation ( <https://ec.europa.eu/chafea/health/funding/joint-actions/documents/ja-2019-presentation-01_en.pdf>)
15. Health Plan for Catalonia 2016-2020. (Department of Health. Catalonia health plan for 2015–2020 (in Catalan). 2016. Available from: <http://salutweb.gencat.cat/ca/el_departament/Pla_salut/pla-de-salut-2016-2020/>.
16. Sustainable Integrated Care Models for Multi-morbidity Delivery, FInancing and Performance (SELFIE). Available from: (<https://www.selfie2020.eu/#artikel>)
17. ACT scale. Experiences from successful real life deployment services using a Collaborative methodology – Plan Do Study Act (PDSA) (https://www.act-at-scale.eu/)
18. NEXTCARE – Personalized care of the chronic patient in a digital health framework (<https://eurecat.org/en/portfolio-items/nextcare/>)
19. Health Plan for Catalonia 2011-15. (Department of Health. Catalonia health plan for 2016–2020 (in Catalan). (in Catalan). 2019. Available from: <http://salutweb.gencat.cat/web/.content/_departament/pla-de-salut/Plans-de-salut-anteriors/Pla-de-salut-de-Catalunya-2011-2015/pla-de-salut-2011-2015/documents/health_plan_english.pdf>.
20. [Agència de Qualitat i Avaluació Sanitàries de Catalunya (AQuAS)](http://aquas.gencat.cat/ca/). <http://aquas.gencat.cat/ca/inici/index.html>
21. Shepperd S, et al. Admission avoidance hospital at home. CochraneDatabaseofSystematicReviews 2016, Issue 9. Art. No.: CD007491. DOI: 10.1002/14651858.CD007491.pub2.
22. Gonçalves-Bradley DC, et al. Early discharge hospital at home. CochraneDatabaseofSystematicReviews 2017, Issue 6. Art. No.: CD000356. DOI: 10.1002/14651858.CD000356.pub4
